# Benchmarking 2D Multi-Object Detection and Tracking Algorithms in Autonomous Vehicle Driving Scenarios

**DOI:** 10.3390/s23084024

**Published:** 2023-04-16

**Authors:** Diego Gragnaniello, Antonio Greco, Alessia Saggese, Mario Vento, Antonio Vicinanza

**Affiliations:** Department of Information and Electrical Engineering and Applied Mathematics, University of Salerno, 84084 Fisciano, Italy; digragnaniello@unisa.it (D.G.); agreco@unisa.it (A.G.); asaggese@unisa.it (A.S.); mvento@unisa.it (M.V.)

**Keywords:** multiple object tracking (MOT), autonomous vehicle driving, deep learning, BDD100K

## Abstract

Self-driving vehicles must be controlled by navigation algorithms that ensure safe driving for passengers, pedestrians and other vehicle drivers. One of the key factors to achieve this goal is the availability of effective multi-object detection and tracking algorithms, which allow to estimate position, orientation and speed of pedestrians and other vehicles on the road. The experimental analyses conducted so far have not thoroughly evaluated the effectiveness of these methods in road driving scenarios. To this aim, we propose in this paper a benchmark of modern multi-object detection and tracking methods applied to image sequences acquired by a camera installed on board the vehicle, namely, on the videos available in the BDD100K dataset. The proposed experimental framework allows to evaluate 22 different combinations of multi-object detection and tracking methods using metrics that highlight the positive contribution and limitations of each module of the considered algorithms. The analysis of the experimental results points out that the best method currently available is the combination of ConvNext and QDTrack, but also that the multi-object tracking methods applied on road images must be substantially improved. Thanks to our analysis, we conclude that the evaluation metrics should be extended by considering specific aspects of the autonomous driving scenarios, such as multi-class problem formulation and distance from the targets, and that the effectiveness of the methods must be evaluated by simulating the impact of the errors on driving safety.

## 1. Introduction

The algorithms that allow a drive-by-wire electric vehicle to navigate autonomously on the road are able to analyze in real-time the data collected from inertial navigation system (INS), cameras, lidars, radars and sonars (perception), planning the trajectory according to the destination to be reached, the rules of the road and the current traffic situation (motion planning) and consequently control the speed and steering of the vehicle (vehicle control). Ideally, the vehicle should move in a smart road, where all the objects on the road, including other vehicles, are interconnected and exchange information with each other; there are preliminary studies in the field of connected autonomous vehicles [1,2,3], but the roads are not yet equipped to accommodate these technologies and, therefore, we focus on autonomous driving vehicles that are not connected with the road and can perceive the environment only relying on their exteroceptive sensors.

The design of the algorithms for autonomous vehicle driving (AVD) is anything but simple and their typologies can be classified into two macro-categories: end-to-end or modular systems [4]. The former is an emerging trend of recent years [5], which requires the training of neural networks with the data collected by the sensors annotated with the corresponding steering and acceleration or braking actions performed by the human driver; according to this paradigm, end-to-end algorithms should learn to imitate the behavior of the human driver (imitation learning or behavior cloning) in presence of the same perceptive stimuli. Recent works [6,7,8] have shown that the performance obtained by these algorithms is far from ensuring safe driving and can be improved only by collecting an enormous amount of data not currently available; moreover, the behavior of end-to-end systems cannot be easily explained and it is difficult to intervene for improving their performance, being monolithic. On the other hand, the modular systems are designed to perform the tasks of perception, motion planning and vehicle control with different modules [9]. These algorithms are preferred today because the outputs of the various processing steps are more controllable and because the modules require less training data than those that are necessary for an effective end-to-end system. Therefore, the focus of this paper is on modular systems and, in particular, on the perception module.

Commercial systems, such as NVIDIA Drive, offer great support to modular systems, providing tools for sensor calibration and data synchronization, ego-motion estimation, and training and acceleration of neural networks for image processing. However, the success of modular algorithms mainly depends on their accuracy in detecting and tracking objects of interest (vehicles and pedestrians) [10,11,12]. In fact, although the motion planning algorithms of self-driving vehicles are able to complete their mission with the use of the inertial navigation system in the absence of obstacles (on a track or on an empty road), they need to estimate position, speed and trajectory of pedestrians, bicycles, motorcycles and vehicles (car, bus, truck) when they move on urban roads or highways. Without this capability, the self-driving vehicle is not able, for example, to predict where and how vehicles and pedestrians are going and, therefore, cannot stop so as not to run over pedestrians, follow vehicles in the same lane keeping a safe distance or avoid accidents with other vehicles [13]. Modular autonomous driving algorithms use multi-object detectors to locate objects of interest in the current image, projecting them into the world reference system with the help of distance information provided by lidars or radars. The tracking step, i.e., the estimation of the trajectory of objects of interest over time, allows instead to evaluate the orientation and speed of vehicles and pedestrians, so as to predict their future position and adjust speed and steering of the vehicle accordingly. Starting from these assumptions, it is clear that the accuracy of multi-object detection and tracking algorithms becomes crucial to ensure safe and comfortable driving. For this reason, in this paper we focus on benchmarking multi-object detection and tracking algorithms applied on images acquired on the road with a camera installed on board the vehicle.

Multi-object tracking (MOT) is one of the most challenging problems in the field of computer vision, as the detection and association of objects is made complex, depending on the scene, by appearance variations of the objects caused by sensor noise, pose (rotation, translation, deformation), partial or total occlusions, variable weather or lighting conditions, and so on [14]. The enormous recent advances in technologies based on deep learning have made it possible to achieve such high performance that MOT algorithms can be successfully used in real applications for surveillance, retail and human–robot interaction [15,16]. However, using images acquired in real-time from cameras installed on board the vehicle makes the problem substantially more challenging, since the camera is not static but moving [17]. This aspect makes object association approaches based on intersection over union (IoU overlap tracking) unusable, since the movement of the vehicle invalidates the concept of spatial proximity between consecutive frames. Methods based on motion estimation, such as the Kalman filter, may also have problems in predicting both the apparent motion of stationary pedestrians and vehicles and their real motion on the road; moreover, object association based on motion prediction becomes even more complex for missed detection in some frames due to partial or total occlusions. Camera movement also complicates the problem of object association through appearance-based re-identification [13,18]. In fact, during the journey traveled by the vehicle, pedestrians or other vehicles are framed from different points of view and are not always totally visible. Consider, for example, a vehicle parked on the roadside, which is first fully visible from the rear, then becomes fully or partially visible laterally and gradually disappears from the image [19]; the features learned for re-identification should be so effective to allow a frame-by-frame object association of the same vehicle framed in totally different conditions. These methods of appearance-based object association are also subject to an increase in occlusions due to the rather low position of the camera and the varying conditions of weather and lighting compared to the classic video-surveillance scenarios in which MOT algorithms are typically used. It is thus necessary to analyze the performance of multi-object detection and tracking algorithms in autonomous driving scenarios, so that the issues arising from these challenges can be identified and solved in future research works.

To this aim, Waymo Open Dataset [20] and BDD100K [21] have recently been released and various multi-object tracking methods have been evaluated in such scenarios [13,18,22,23,24]. However, scientific papers published so far have not taken into account all the most recent and popular multi-object detection and tracking algorithms, as well as their combinations. Furthermore, even for the methods already tested, the experimental results were computed not considering all the evaluation metrics proposed in the literature. Moreover, the effect of the detector on the overall performance has not been generally evaluated separately, although it is particularly significant.

Based on these observations, the novel contributions of the paper can be summarized as follows:

- The most promising modern methods for multi-object detection (RetinaNet [25], EfficientDet [26], YOLOv5 [27], YOLOX [28], HRNet [29], Swin Transformer [30], ConvNext [31]) and tracking (DeepSORT [32], UniTrack [33], QDTrack [18]), together with a multi-task approach (FairMOT [34]), have been adapted and fine-tuned for detecting and tracking the objects of interest and evaluated in all their possible combinations, obtaining 22 new MOT algorithms for the context of autonomous vehicle driving.
- The considered multi-object detectors are evaluated separately in terms of Precision, Recall and F-Score, and mIoU by also analyzing Recall and classification accuracy for each class of interest. This analysis, neglected in similar papers, is very useful to determine the impact of this module on the overall performance of the multi-object tracking methods.
- The considered multi-object tracking approaches are evaluated by computing not only the standard CLEAR [35], IDF1 [36] and HOTA [37] metrics, but also considering the recent TETA [23]. In this way, we can separately analyze the influence of the detector and of the data association strategy on the overall performance of the multi-object tracking methods, also with class-agnostic matching strategies.
- The analysis carried out in the proposed experimental framework allows to identify the limitations of the metrics and of the multi-object detection and tracking algorithms applied in the context of self-driving vehicles, as well as the necessity to realize a framework for simulating the impact of perception errors on autonomous navigation, indicating useful future research directions.

The paper is organized as follows. In Section 2, we report a description of the related works. Section 3 describes the proposed experimental framework, giving details on the considered dataset, multi-object detectors, multi-object trackers, evaluation metrics and experimental setup. In Section 4, we report the results in terms of detection, classification and tracking, discussing the main findings and the insights inferred from the experiments in Section 5. Finally, in Section 6 we draw conclusions and discuss the possible future directions of research in this field.

## 2. Related Works

**Figure 1:**
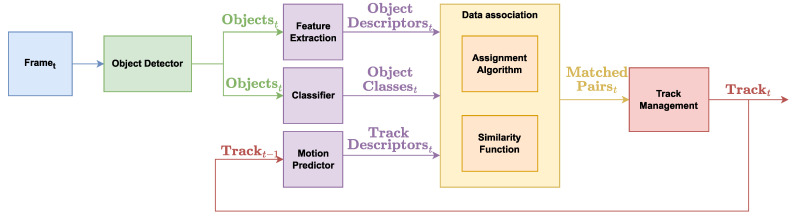
General architecture of a multi-object tracking method. The object detector analyzes the frames localizing the objects. For each object, the feature extraction module extracts the descriptors while the classifier predicts the class. The previously tracked objects are projected in the current frame by the motion predictor. The data association module aims to match the detected objects with the previously tracked ones in order to provide the matched pairs. The similarity function and the assignment algorithm completely characterize the approaches. The former estimates the similarity between tracked and detected objects by using appearance-based and position-based features, while the latter defines a strategy to associate them. Finally, the track management module defines and applies the rules for new and expired objects.

Multi-object tracking algorithms aim to follow the trajectories traveled by the objects of interest (people, vehicles and so on) in different frames of a video sequence [38]. The MOT trends have been recently described in a survey by Guo et al. [39]. The authors classify the methods into three basic groups: tracking-by-detection, joint detection and tracking, and transformer-based tracking. The most used strategy is multi-object tracking-by-detection [40], whose architecture is depicted in Figure 1 and involves three basic steps: (i) multi-object detection [14], which aims to locate all the objects of interest in the scene in each frame, representing them typically by means of bounding boxes (detected objects or targets); (ii) data association [41], which requires the calculation of a similarity measure to perform frame-by-frame re-identification based on information on position and/or appearance and the use of a matching technique to associate the detected objects with those tracked in the previous frames (matched pairs) [42]; track management, which allows to update matched pairs and manage the new objects and the expired objects.

Most of the recent works introduce new algorithms to carry out the various processing steps required for multi-object tracking. In particular, novel methods have been proposed to extract representative descriptors of the objects [18,34] and to improve the re-identification capability of similarity functions [32,33]. Other papers are more focused on the definition of new architectural paradigms [43,44,45]. The methods are typically evaluated with the metrics proposed to analyze their capability to track objects, namely, CLEAR metrics (especially MOTA and MOTP) [35], IDF1 [36], HOTA [37] and TETA [23]. These metrics will be thoroughly described in Section 3.4. However, in most cases, only a subset of the metrics is considered, although they adopt different matching strategies; moreover, the methods are almost always tested on images acquired by fixed cameras and not in road scenarios.

Wang et al. [33] propose UniTrack, which is a task-agnostic appearance-based model learned in a supervised fashion. The model has been tested for multi-object tracking on the MOT16 dataset [46], which only contains people samples acquired from static cameras, in terms of MOTA, HOTA and IDF1. Pereira et al. [47] evaluate SORT [48] and DeepSORT [32] methodologies with different similarity functions on the same dataset by reporting the results in terms of MOTA. StrongSORT, designed by Du et al. [49], improves the MOTA performance achieved by DeepSORT over MOT16 and KITTI [50]. Zhang et al. [34] propose FairMOT, which consists of a single network jointly trained for detection and re-identification. The model is evaluated on MOT16 in terms of MOTA and IDF1. Luiten et al. [37] carry out a comprehensive analysis of multi-object tracking methods but it is limited to MOT20 [51], which is focused on people tracking with fixed cameras. Bergmann et al. [52] adopt the same dataset for testing their Tracktor and Tracktor++ methods; the analysis is limited to MOTA. TransMOT [44], and TrackFormer [45] formulate the tracking problem end-to-end with a transformer-based approach and achieve state-of-the-art performance on MOT16 and MOT20.

A boost to the application of multi-object tracking methods on images acquired by moving cameras installed on board vehicles has been given by the two recent challenges proposed on Waymo Open Dataset [20] and on BDD100K [21]. In the former challenge, the competing methods are evaluated in terms of MOTA and MOTP; in the latter, various metrics inspired by MOTA, HOTA and IDF1 and adapted to a multi-class multi-object tracking problem are adopted to draw up the ranking of the competition. In both cases, the comparison of the methods is limited to the leaderboard and the organizers do not discuss the results of the competing approaches. Therefore, the few experimental evaluations available on these datasets are reported by the authors who decided to use Waymo Open Dataset and BDD100K for their experiments.

Lu et al. [13] evaluate RetinaTrack, a joint model for multi-object detection and tracking based on RetinaNet, on the Waymo Open Dataset in terms of MOTA. The comparison is limited to a few models, such as Tracktor and Tracktor++. A comparison between several approaches on the BDD100K dataset can be found in QDTrack [18] and ByteTrack [22]. In both cases, the results are limited to the methods available in the leaderboard of the challenge, which do not completely represent the state of art methodologies; furthermore, the results are reported in terms of mMOTA and IDF1 without considering more recent metrics, such as HOTA and TETA. In more detail, Pang et al. [18] propose a similarity learning approach for the extraction of an appearance-based representation for object re-identification along the frames; the approach demonstrates a remarkable accuracy in the experiments performed on BDD100K. Zhang et al. [22] propose an association method, named BYTE, for the association of high-score and low-score detection boxes. Their method, namely ByteTrack, achieves state-of-the-art performance on different tracking benchmarks, such as MOT20 and BDD100K. In addition, MOTR [43] has been compared only with the methods submitted in the leaderboard of BDD100K challenge. Unicorn, proposed by Yan et al. [24], is a single neural network for dealing with various tracking problems. It has been evaluated on BDD100K but the experiments are limited to the metrics adopted in the challenge. Finally, Li et al. [23] propose TETer, a new tracking methodology based on the concept of class-agnostic association. The comparison is performed with a limited set of methods (QDTrack [18] and DeepSORT [32]) and metrics (TETA, MOTA, IDF1).

From the analysis of the related works, a lack of comprehensive experimentation of recent multi-object tracking methods on road scenarios, as well as a partial use of the available metrics, emerges. Furthermore, few combinations of recent detectors and data association methods have been tested in the field of autonomous driving. In addition, the contribution of multi-object detectors is never evaluated separately with metrics fit for the purpose (Precision, Recall, F1-Score, mIoU, Recall for each class); therefore, it is difficult to infer which architectural element of the multi-object tracking method provides a decisive boost to performance. The experimental analysis proposed in this paper aims to fill these gaps, analyzing 22 different approaches in road scenarios to find out their strengths and limitations.

## 3. Experimental Framework

The goal of this paper is the design of an experimental framework to evaluate the performance of multi-object tracking methods applied to sequences of images acquired on board the vehicle, using different types of modules that constitute the MOT approaches. The methods always include a multi-object detector able to locate the objects in the scene and, typically, to predict the class of the detected objects. The temporal analysis of the objects is performed by the data association module, which exploits appearance-based (object descriptors) and position-based (track descriptors obtained through motion prediction) information to compute similarity scores between detected and previously tracked objects. The assignment algorithm optimizes someway the matched pairs by analyzing the similarity scores. Finally, the track management module defines and applies the rules for new and expired objects. We generalize the architecture of MOT approaches and realize a framework in which we evaluate 22 different multi-object tracking approaches, by combining seven multi-object detectors with three tracking (data association and track management) approaches plus a multi-task method. The 22 algorithms are trained and tested on the same dataset and evaluated with the same metrics in order to draw fair conclusions.

In the next subsections, we describe the dataset adopted for our experiments (Section 3.1), the considered multi-object detectors (Section 3.2) and multi-object tracking approaches (Section 3.3), the adopted evaluation metrics (Section 3.4) and the experimental setup that we implemented for carrying out the benchmark analysis (Section 3.5).

### 3.1. Dataset

**Figure 2:**
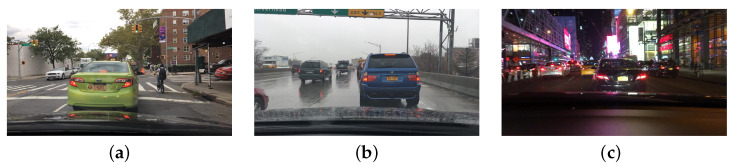
Samples from the BDD100K validation set. The dataset has been collected with different illumination and weather conditions. (**a**) Daylight cloudy. & (**b**) Daylight rainy. & (**c**) Night.

A dataset suitable for training and evaluating multi-object tracking methods applied in self-driving vehicles must contain several video sequences acquired by a camera installed on board the vehicle in various weather and lighting conditions, as shown in Figure 2.

**Figure 3:**
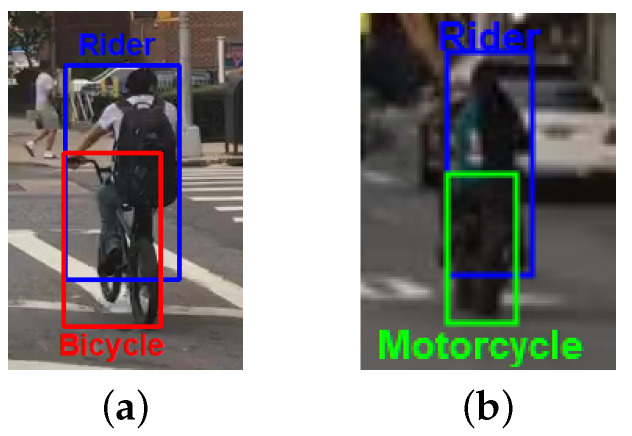
Examples of a bicycle and a motorcycle and their riders from the BDD100K dataset. (**a**) Bicycle and Rider. (**b**) Motorcycle and Rider.

In addition, this dataset should be annotated frame by frame with the bounding boxes of all the objects with class and identifier labels; with this information, it is possible to train a multi-object detector able to recognize the classes of interest and a feature extraction method for appearance-based data association, as well as to evaluate the performance of a multi-object tracking algorithm. For this reason, our choice fell on BDD100K [21], which is the largest publicly available dataset for multi-object tracking applied to autonomous driving scenarios. It consists of 100,000 videos acquired in several scenarios, covering a large set of weather and lighting conditions, with a camera installed on board a vehicle. Among them, 2000 videos 40 s long each, are annotated for multi-object tracking at 5 fps, so the frames annotated are approximately 200 for each video (around 398,000 frames are labelled). The bounding boxes in the video are around 3.3 million and the total number of subjects is around 130,600. The available classes for each bounding box are pedestrian, rider, car, truck, bus, train, motorcycle, bicycle, traffic light and traffic sign; as was done in the BDD100K challenge, we consider only moving objects, so we exclude from our experiments the last two categories. In addition, we do not consider trains, since there are only dozens of instances of this class, often wrongly annotated. Thus, we take into account seven classes of interest. As shown in Figure 3, bicycle and motorcycle represent only the conveyance without the person, while the driver is categorized as rider.

The dataset is already partitioned into training, validation and test sets with the following percentages: 70% training (1400 videos), 10% validation (200 videos) and 20% test (400 videos). For our experiments, we used the training set for the fine-tuning of the object detectors described in Section 3.2, while we adopted the validation set for the experimental evaluation of the various components of the multi-object tracking algorithms; the test set is excluded because its annotations are not publicly available.

We preferred BDD100K to Waymo Open Dataset [20], which is also acquired in autonomous driving scenarios, for the following reasons. First of all, BDD100K contains almost double the annotated video sequences and frames available in Waymo Open Dataset. The latter includes more object bounding boxes, being often recorded in crowded scenarios, but BDD100K is acquired in a wider range of environments, weather and lighting conditions. In addition, Waymo Open Dataset provides only four object categories (pedestrian, vehicle, cyclist, sign), definitely fewer than the seven classes we can consider in BDD100K. Since the focus of this paper is to evaluate the robustness of multi-object tracking methods in road driving scenarios, BDD100K is the most suitable choice; Waymo Open Dataset would be a better choice for testing autonomous driving algorithms, since it provides high-quality multi-sensor data useful for autonomous navigation.

### 3.2. Object Detectors

Although this module is rather underestimated in multi-object tracking methods, multi-object detectors have a fundamental impact on the performance of MOT algorithms, as we will experimentally demonstrate in Section 4.1. The choice of multi-object detector is often made superficially, paying more attention to appearance descriptors and data association strategies. The performance of this module is not evaluated separately from the other components of a MOT algorithm, despite the fact that experiments with an oracle multi-object detector (groundtruth bounding boxes used as detector output) demonstrated that the impact of detection errors on the overall performance is significant [18]; consequently, the experiments are carried out by fixing an object detector, which becomes an integral part of the multi-object tracking method. This approach is not optimal, since modern multi-object detectors contain various architectural elements that can be suited to mitigate some of the typical problems of the road driving scenarios (occlusions, objects of different sizes at different distances, multi-class classification, efficiency) and their peculiarities may not be equally suited for all the data association strategies. In our experimental analysis, we want to pay attention to this aspect; thus, we selected some modern multi-object detectors based on the characteristics that could be suitable for road driving scenarios.

The chosen multi-object detectors cover a large set of approaches from the standard convolutional neural networks to the recent vision transformers, considering anchor-based and anchor-free methods and loss functions designed to deal with unbalanced datasets. In particular, we selected and fine-tuned with the BDD100K training set the following multi-object detectors: RetinaNet [25], EfficientDet [26], YOLOv5 [27], YOLOX [28], FairMOT [34], HRNet [29], Swin Transformer [30] and ConvNext [31]. Some of them [29,30,31] are at the top of the BDD100K leaderboard. We chose a set of object detectors with architectures that are designed for exploiting different aspects of an autonomous driving scenario. RetinaNet [25] deals with class imbalance, EfficientDet [26] and YOLOv5 [27] face up objects with different sizes, YOLOX [28] has a better management of occlusions and FairMOT [34] points to the computational efficiency, while HRNet [29], Swin Transformer [30] and ConvNext [31] achieve state-of-the-art performance on the chosen dataset for the detection task. Detailed descriptions of all the methods and comprehensive explanations of the reasons that brought us to choose them are listed in the following.

RetinaNet [25] is a multi-class multi-object detector that is trained with the focal loss to mitigate the imbalance of performance on different object classes when the dataset is highly unbalanced. With the problem of interest being multi-class and considering that BDD100K is very unbalanced, the learning procedure used in RetinaNet could guarantee higher accuracy than other methods on less-represented classes.

EfficientDet [26] is a multi-object detector based on the EfficientNet backbone [53] that uses the bi-directional feature pyramid network (BiFPN) to combine the features extracted at different scales. Its multi-resolution feature extraction can be suited for road driving scenarios, where objects at different distances from the camera may have various sizes. In addition, its more efficient architecture compared to other detectors may allow it to achieve high processing speed.

YOLOv5 [27] is an object detection model inspired by YOLOv4 [54] that adopts CSPDarknet-53 as backbone and several modern techniques for fostering training convergence and improving inference accuracy. The main novelty introduced by YOLOv5 is the anchor box selection process, which automatically allows to learn the best anchor boxes for the specific training set. Considering that the multi-object detector has to localize objects of different shapes (pedestrians, motorcycles, vehicles, trucks), often partially occluded, this feature can be very effective for the problem at hand.

YOLOX [28] is based on YOLOv3 [55] but it introduces various novelties. First of all, it adopts a center-based anchor-free approach and a mosaic augmentation which may be very suitable for dealing with partial occlusions. In addition, YOLOX has decoupled heads for regression and classification to solve the problem of spatial misalignment of the features necessary for the two tasks; this peculiarity may be useful for dealing with multi-object detection and classification in crowded roads.

FairMOT [34] is a single multi-task network trained for performing multi-object detection and description. The fusion in a single neural network of the two tasks allows an improvement of the inference speed. It uses an anchor-free approach based on CenterNet with ResNet-34 (DLA version) as backbone, so it may be effective with partial occlusions. We will test the FairMOT multi-object tracking approach described in Section 3.3.

High-Resolution Network (HRNet) [29] has the objective to maintain high-resolution representations throughout the whole feature extraction procedure. This goal is achieved by connecting several high-to-low resolution convolution streams in parallel and by exchanging information across resolutions. In this way, the obtained representation is spatially more precise and the latent space maintains rich semantic information. Thanks to these architectural peculiarities, HRNet achieved remarkable semantic segmentation results in road urban scenarios; we expect that the same can happen for object detection in similar conditions.

Swin Transformer (SwinT) [30] is a transformer-based neural network that is achieving state-of-the-art performance in various object detection tasks. Its success is mainly due to hierarchical feature maps and patch merging that foster scale invariance and the limitation of self-attention to non-overlapping local windows while allowing for cross-window connections, which enforces shift invariance. These features can definitely be relevant even in the considered autonomous driving scenarios.

ConvNext [31] is a pure convolutional neural network obtained by adding to a standard ResNet modern components that contribute to favorably compete with transformer-based models. Thanks to these training techniques and architectural upgrades (inverted bottlenecks, large kernels, GELU, Layer Normalization, fewer activation functions and normalization layers, separate downsampling layers), it achieved 87.8% ImageNet top-1 image classification accuracy and outperformed Swin Transformer in object detection over COCO dataset. Therefore, we also consider this multi-object detector promising for the problem at hand.

### 3.3. Object Tracking

The tracking methods define the data association strategy, which includes the assignment algorithm and the similarity function. The choice of the latter determines how to compute the object descriptors and the track descriptors, namely, the feature extraction method for the appearance-based representation and the motion prediction algorithm, as well as the combination rule for computing the similarity. As was already done for multi-object detectors, we choose tracking methods that have different characteristics, especially in terms of data association strategies. In particular, the considered multi-object tracking methods are: DeepSORT [32], UniTrack [33], FairMOT [34] and QDTrack [18]. The chosen trackers consider positional-based and appearance-based similarity functions and association strategies that can be effective in different situations in a road scenario. DeepSORT [32] introduces a combination of positional and appearance features that strongly matches the tracklets in case of short movements, while UniTrack [33] gives more importance to the appearance; that is indeed the only information which QDTrack [18] considers for carrying out the association between detected and previously tracked objects. On the other hand, FairMOT [34] provides a computationally efficient way to extract features for object association. In the following, we provide a detailed description of the chosen tracking methods and further motivations for our choice to adopt them.

DeepSORT [32] is an advanced version of SORT [48] that uses a similarity function based on the combination between the position-based similarity provided by SORT and an appearance-based similarity. The position-based similarities are computed as the Mahalanobis distances between the centers of the bounding boxes of the detected objects and the position estimated with Kalman filter for the previously tracked objects. The appearance-based similarities are the cosine distances between the representations of the detected objects and their stored representations. The object representation is computed with a convolutional neural network with two convolutional layers and six residual blocks. The matched pairs are then obtained with a two-step matching cascade technique. In the first step, the weighted sum between the two similarities is used for matching pairs with the Hungarian algorithm, which solves a linear assignment problem. In the second step, the remaining objects are matched again with the Hungarian algorithm, but applied only on the position-based similarities. The track management algorithm deletes unmatched tracked objects after a configurable number of frames, while detected objects not matched are considered new objects to track.

UniTrack [33] is similar to DeepSORT as it adopts the same two-step matching with the Hungarian algorithm, but the object description, the appearance-based similarity and the track management are different. In particular, the object representation is based on ResNet-18 or ResNet-50 (we adopted the latter for our experiments) and the similarity function is computed through the reconstruction similarity metric (RSM), which builds an affinity matrix to extract the reconstruction matrices that are used to build the representation for matching the pairs. The track management strategy removes the unmatched tracked objects after 1 s, while new objects are tracked after two consecutive frames in which they appear and are matched.

FairMOT [34] adopts the same two-step matching with Hungarian algorithm and track management technique used in DeepSORT, but the appearance-based similarity is totally different. In fact, FairMOT computes the object descriptor with a branch of its multi-object detector. This branch is a convolutional layer with 128 kernels applied on top of the backbone features. Bi-directional softmax is adopted as similarity function.

QDTrack [18] uses only an appearance-based similarity. The object descriptor is obtained with a convolutional neural network with four convolutional blocks, trained through a contrastive learning approach that determines the feature representation, allowing to perform an effective re-identification. A quasi-dense similarity learning is applied to look at the previously tracked objects and to find the best matching by using a bi-directional softmax as similarity function. The matched pairs are not obtained by solving a linear assignment problem, but by selecting the pairs corresponding to the maximum similarity. The track management algorithm removes expired objects after a configurable number of frames, while it treats differently the new objects according to their bi-softmax score. In particular, detected objects with a bi-softmax score higher than a confidence threshold are immediately tracked, while the others are considered after a configurable number of consecutive frames in which they are matched with a lower bi-softmax score.

### 3.4. Evaluation Metrics

In the following subsections, we describe the metrics used to evaluate multi-object tracking methods in terms of detection (Section 3.4.1), classification (Section 3.4.2) and tracking (Section 3.4.3) capabilities.

#### 3.4.1. Detection

To compute the performance of a multi-object detection method, it is necessary to define the sets of true positives (TP), false positives (FP) and false negatives (FN). In fact, the metrics defined to evaluate these methods are based on the number of true positives (|TP|), false positives (|FP|) and false negatives (|FN|).

With Pred being the set of bounding box predictions, Gt the set of bounding boxes in the groundtruths and (p,gt) with p∈Pred and gt∈Gt, an association between a prediction and a groundtruth box on the same frame, the sets of true positives, false positives and false negatives are composed according to the Jaccard criteria:

(1) TP={(p,gt)|p∈Pred∧gt∈Gt∧IoU(p,gt)≥α}

(2) FN={gt|gt∈Gt,∄p∈Pred:(p,gt)∈TP}

(3) FP={p|p∈Pred,∄gt∈Gt:(p,gt)∈TP}

where IoU(p,gt) is the intersection over union computed between the bounding boxes of a prediction p∈Pred, and of a groundtruth element gt∈Gt and α is the detection threshold, used to determine whether the association is accurate enough to consider it a true positive.

Adopting these sets for TP, FP and FN, the detection results are evaluated in terms of the standard Precision (Pr), Recall (Re) and F1-score (F1) metrics, defined as follows:

(4) $$Pr=\frac{\left|TP\right|}{\left|TP|+|FP\right|}$$

(5) $$Re=\frac{\left|TP\right|}{\left|TP|+|FN\right|}$$

(6) $$F1=2\cdot \frac{Pr\cdot Re}{Pr+Re}$$

Precision measures the capability of the approach to reject false positives, which could cause sudden vehicle decelerations and steering. Recall evaluates the sensitivity of the method to detect the objects of interest, and is very important as false negatives could cause vehicle collisions with objects in the scene. The F1-score is the harmonic mean between the two values.

The precision of the bounding box around the detected object can be a crucial factor for data association; in fact, an error in the alignment of the bounding box around the detected object can cause the extraction of an object descriptor that is not representative enough. Therefore, we measure this alignment in terms of mIoU on the detected objects as follows:

(7) $$mIoU=\frac{\sum_{TP}IoU(p,gt)}{\left|TP\right|}$$

The higher is the mIoU value, the better is the alignment of the bounding box of detected objects with the groundtruth annotations.

In addition, we will also compute the Recall for each class in order to have information about the detection accuracy on specific object classes. With *c* being the generic class, we can define the sets $TP_{c}$ and $FN_{c}$ as follows:

(8) $$TP_{c}=\left\{\left(p,gt\right)\in TP\wedge Class\left(gt\right)=c\right\}$$

(9) $$FN_{c}=\left\{gt|gt\in Gt\wedge Class\left(gt\right)=c,∄p\in Pred:\left(p,gt\right)\in TP\right\}$$

Therefore, the Recall $Re_{c}$ for each class *c* can be computed as follows:

(10) $$Re_{c}=\frac{|TP_{c}|}{|TP_{c}|+|FN_{c}|}$$

The higher the $Re_{c}$, the better is the accuracy of the method in detecting objects of class *c*.

#### 3.4.2. Classification

We find it useful to compute the classification accuracy to determine the amount of possible associations made between objects of different classes. In fact, these errors may lead to incorrect driving actions, as the behavior of the driver should certainly be different when encountering pedestrians than when encountering vehicles.

The set of correct classifications CC can be defined as follows:

(11) CC={(p,gt)∈TP∧Class(p)=Class(gt)}

In addition, the set of correct classifications $CC_{c}$ for a specific class *c* is:

(12) $$CC_{c}=\left\{\left(p,gt\right)\in TP_{c}\wedge Class\left(p\right)=c\right\}$$

We compute the overall classification accuracy *A* as follows:

(13) $$A=\frac{\left|CC\right|}{\left|TP\right|}$$

In addition, the classification accuracy $A_{c}$ for a specific class *c* is:

(14) $$A_{c}=\frac{|CC_{c}|}{|TP_{c}|}$$

#### 3.4.3. Tracking

All the metrics proposed in the literature for evaluating multi-object tracking algorithms are based on the definition of true positives (TP), false positives (FP) and false negatives (FN). However, unlike multi-object detection, TP, FP and FN sets are built with an optimization process that typically maximizes the value of the specific tracking metric. Therefore, for each metric it is first necessary to describe the method by which the aforementioned sets are built and then the definition of the metric can be provided. In the following, we describe the most popular metrics proposed so far, namely, CLEAR [35], IDF1 [36], HOTA [37] and TETA [23], which we adopted to evaluate the performance of the multi-object tracking methods.

CLEAR metrics [35] include Identity Switch (IDSW), Multiple Object Tracking Accuracy (MOTA) and Multiple Object Tracking Precision (MOTP). IDSW is the number of association errors between two consecutive frames; each of these events happens when two consecutive instances of the same detected object are tracked with two different identifiers. The TP set is optimized with a two-step method. In the first step, the matching is performed with couples (p,gt) for which the similarity value is higher than a configurable threshold and that do not cause an identity switch. In the second step, the Hungarian algorithm selects the set of remaining matches that maximize as a primary goal |TP| and as secondary goal the mean similarity across TP. Then, FN and FP are built as shown in Equations (2) and (3).

MOTA measures the multi-object tracking capability without distinguishing between detections and associations, as follows:

(15) $$MOTA=1-\frac{\left|FN|+|FP|+|IDSW\right|}{\left|TP|+|FN\right|}$$

where |IDSW| is the number of identity switches, and |TP|+|FN| corresponds to the number of groundtruth instances.

MOTP measures the matching error as the average of the similarities S(p,gt) computed over all the true positive samples.

(16) $$MOTP=\frac{\sum_{TP}S(p,gt)}{\left|TP\right|}$$

Identity F1-score (IDF1) [36] is a metric computed by performing the matching for TP, FP and FN at trajectory level and not at detection level. Thus, the definition is based on three sets: Identity True Positives (IDTP), Identity False Negatives (IDFN) and Identity False Positives (IDFP). The optimization procedure adopted to build these sets has the goal of minimizing the sum of |IDFN| and |IDFP|. In particular, the Hungarian algorithm is used to select the trajectories to match (to build IDTP) by finding the maximum number of associations whose similarity is higher than a configurable threshold.

According to these definitions of IDTP, IDFN and IDFP, Identity Precision (IDP), Identity Recall (IDR) and Identity F1-score (IDF1) are computed as follows:

(17) $$IDP=\frac{\left|IDTP\right|}{\left|IDTP|+|IDFP\right|}$$

(18) $$IDR=\frac{\left|IDTP\right|}{\left|IDTP|+|IDFN\right|}$$

(19) $$IDF1=\frac{\left|IDTP\right|}{\left|IDTP|+0.5|IDFN|+0.5|IDFP\right|}$$

Higher Order Tracking Accuracy (HOTA) is a metric that separately considers detection and data association capabilities by measuring them with two different indices, namely, DetA and AssA. Both terms are dependent on the definition of TP, FP and FN, which is similar to the procedure applied for CLEAR metrics. The Hungarian algorithm determines the set of matches, maximizing as a first goal |TP|, as a secondary goal the mean of the association similarities across TP, and as a third goal the mean of the localization similarities across TP. The matching is performed only if IoU(p,gt) is higher than a configurable threshold. The final metrics consist of the area under the curve obtained evaluating the various optimization solutions at different localization thresholds.

According to this definition of TP, FP and FN, the detection accuracy $DetA_{\alpha }$ for a specific localization threshold α is computed as follows:

(20) $$DetA_{\alpha }=\frac{\left|\mathrm{TP}\right|}{\left|\mathrm{TP}|+|\mathrm{FN}|+|\mathrm{FP}\right|}$$

The association accuracy AssA depends on sets that we can define for p|(p,gt)∈TP:

- TPA(p): True Positive Associations, the set of true positives whose predicted and groundtruth identifier is the same of *p*.
- FNA(p): False Negative Associations, the set of true positives whose groundtruth identifier is the same of *p* but with a different predicted identifier.
- FPA(p): False Positive Associations, the set of true positives whose predicted identifier is the same of *p* but with a different groundtruth identifier.

For each p|(p,gt)∈TP, it is possible to define the association score as follows:

(21) $$𝒜\left(p\right)=\frac{\left|\mathrm{TPA}(p)\right|}{\left|\mathrm{TPA}(p)|+|\mathrm{FNA}(p)|+|\mathrm{FPA}(p)\right|}$$

Therefore, the association accuracy $AssA_{\alpha }$ for a specific localization threshold α can be computed as the average of the association scores:

(22) $${\mathrm{AssA}}_{\alpha }=\frac{1}{\left|\mathrm{TP}\right|}\sum_{p\in TP}𝒜\left(p\right)$$

Finally, $HOTA_{\alpha }$ for a specific localization threshold α is defined as the geometric mean between $DetA_{\alpha }$ and $AssA_{\alpha }$:

(23) $$HOTA_{\alpha }=\sqrt{DetA_{\alpha }\cdot AssA_{\alpha }}$$

CLEAR, IDF1, and HOTA metrics are designed for single-class multi-object tracking. When they are applied to multi-class multi-object tracking, their values are computed separately on objects of each class (considering the groundtruth label), associating only the pairs (p,gt) for which Class(p)=Class(gt). The final value is then obtained by computing the average of the values obtained on the single classes, as done for the BDD100K challenge. Therefore, we compute mMOTA, mMOTP, mIDP, mIDR, mIDF1, massA, mDetA and mHOTA metrics in this way.

TETA [23] was instead designed to be applied directly to multi-class multi-object tracking. It adopts a class-agnostic association method, so it is not separately computed on the objects of each class, since all the objects are considered together for the association independently of the class. In this way, the measured multi-object tracking performance is not affected by the classification accuracy. TETA depends on three contributions, namely, localization accuracy (LocA), association accuracy (AssocA) and classification accuracy (ClsA), which depends in turn on the definition of TP, FN and FP sets. The optimization of these sets is performed with the Hungarian algorithm as in HOTA, but it is class-agnostic and aims to maximize LocA and AssocA.

In particular, LocA and AssocA measure the localization and association accuracy, respectively. They are computed with the same procedure defined in HOTA for DetA and AssA, but the associations are class-agnostic.

ClsA measures the classification accuracy and depends, for a given class *c*, on:

(24) TPC(c)={(p,gt)∈TP∧Class(p)=c∧Class(gt)=c}

(25) FNC(c)={(p,gt)∈TP∧Class(p)≠c∧Class(gt)=c}

(26) FPC(c)={(p,gt)∈TP∧Class(p)=c∧Class(gt)≠c}

With these sets computed for all the classes, ClsA is obtained as follows:

(27) $$ClsA=\frac{\left|TPC\right|}{\left|TPC|+|FNC|+|FPC\right|}$$

Finally, TETA is the arithmetic mean of LocA, AssocA and ClsA:

(28) $$TETA=\frac{LocA+AssocA+ClsA}{3}$$

### 3.5. Experimental Setup

With the dataset, the detection and tracking approaches and the evaluation metrics defined, we can now describe the setup of the experimental framework that allows us to use the BDD100K dataset to train and evaluate the 22 multi-object tracking methods, computing all the evaluation metrics defined in terms of detection, classification and tracking. To build the framework, we used the tools already available for the BDD100K challenge [21] and in TrackEval [56], adapting them to our purposes and adding the missing features.

First of all, to make possible the composition of methods with different multi-object detectors and trackers, we decoupled the dependencies of the two modules by defining a common and generic interface for each tracking method. In this way, we can carry out separately the two steps, ensuring that the multi-object detectors produce only the outputs with the defined format.

Therefore, for the detection step the only constraint is that the data format is compliant with the BDD100K challenge framework [21]. In particular, the multi-object detectors must produce as output a list of predictions obtained when they are applied on the BDD100K validation set. Each prediction is characterized by the following fields: filename, video identifier, frame identifier, bounding box (xywh format), confidence score and category identifier (pedestrian, rider, car, truck, bus, motorcycle, bicycle). We trained the seven multi-object detectors (RetinaNet, EfficientDet, YOLOv5, YOLOX, HRNet, SwinT, ConvNext) and FairMOT by using the BDD100K training set, considering only the seven classes of interest. The results of all the detectors are then parsed to produce the output compliant with the defined data format. The framework offers the possibility to run all the multi-object detectors on the images of the BDD100K validation set, store the outputs and compute all the detection (Precision, Recall, F1-score, mIoU, Recall for each class) and classification (overall and by class accuracy) metrics. This structure allows to run all the methods once, producing the outputs in a format compatible with all the tracking algorithms, and to easily integrate new multi-object detectors.

The tracking step firstly requires the initialization of the detection results, which are loaded and re-arranged to extract information regarding the specific video and frame. Then, data association and track management procedures required by the various tracking algorithms are executed. We integrated in this framework the considered trackers, namely, DeepSORT, UniTrack, FairMOT and QDTrack, in order to make them compliant with the defined data format. Each method produces, for each video, a JSON file of the results that is compliant with the TrackEval standard [56]. Most of the considered tracking metrics (mMOTA, mMOTP, IDSW, mIDF1, mIDP, mIDR, mHOTA, mDetA, mAssA, TETA, LocA, AssocA, ClsA) are already available in TrackEval, while we implemented the missing ones. The framework obviously takes into account the differences among the matching techniques adopted for the various metrics.

## 4. Results

In the following subsections, we report and comment detection (Section 4.1), classification (Section 4.2) and tracking results (Section 4.3) achieved by the considered methods.

### 4.1. Detection Results

The results achieved by the considered multi-object detectors are reported in Table 1. We can observe a substantial balance in terms of F1-score between the top five methods, namely, SwinT, ConvNext, HRNet, YOLOX and YOLOv5. SwinT is slightly higher (0.78), as it reaches the best trade-off between Precision (0.73) and Recall (0.82). ConvNext is slightly more effective in terms of Recall (0.83), while Precision is the prerogative of YOLOX (0.75), HRNet (0.74) and YOLOv5 (0.74). We expect methods with higher Recall to be less prone to possible collisions with undetected objects, while those with higher Precision will cause fewer unnecessary decelerations and steering due to false positives.

This balance is also evident in terms of mIoU, whereby all the above methods obtain the same value (0.84), except HRNet (0.83); we can therefore expect that the latter may be less effective in data association, due to a higher misalignment of the bounding boxes. The gap in terms of F1-score between the top five methods and the others, namely, FairMOT (0.70), EfficientDet (0.67) and RetinaNet (0.65), is quite evident. FairMOT achieves the top Precision (0.93), but also the worst Recall (0.56), proving to be a rather cautious multi-object detector; the worst result in terms of mIoU (0.80) also implies a possible reduction in the capability to extract an effective object descriptor, even if this neural network produces the representation used for data association with its own branch. EfficientDet shows a good trade-off between Precision (0.71) and Recall (0.64), but the latter value is too low compared to the average; in terms of mIoU, the result is slightly lower (0.82) than HRNet. RetinaNet achieves a better Recall (0.74) than the last two methods, but the worst performance in terms of Precision (0.58); the high amount of false positives may cause problems for the autonomous driving algorithm, while the result in terms of mIoU (0.81) is in the middle between EfficientDet and RetinaNet.

Observing the performance in terms of Recall for each class, we can see a clearer gap between the top three methods, i.e., SwinT, ConNext and HRNet, and the others; we expect that this difference will also be evident in tracking metrics that consider the class to make the associations. As for the Car class, YOLOX (0.83) and YOLOv5 (0.81) manage to keep up with the best methods (0.84–0.85), while RetinaNet (0.77), EfficientDet (0.69) and FairMOT (0.60) are more distant. On the other classes, the first three methods outperform YOLOX by at least four percentage points, YOLOv5 even more and the last three by a long way. RetinaNet deserves a special mention, as the optimization through focal loss allows it to obtain a more balanced performance on the various classes and a smaller gap compared to the best approaches on the less represented classes.

However, the superiority of the top three methods is evident and they should be preferred as multi-object detectors in the field of self-driving vehicles; nevertheless, to the best of our knowledge, these methods had never been tested together with state-of-the-art tracking algorithms on image sequences acquired from cameras installed on board a vehicle.

### 4.2. Classification Results

The association between detected objects and groundtruth elements for evaluating multi-object detection results is class-agnostic, i.e., no class matching is evaluated (see Equation (1)). In this way, we evaluated in the previous Section the localization capability of the multi-object detectors, while we separately analyzed their object classification capability. The results of this experiment are reported in Table 2.

Analyzing these results, we can notice a substantial overall balance, considering that the average accuracy is equal to 0.97 for all methods and 0.96 for RetinaNet; for the latter, the result is in contrast with respect to what was observed for the detection and seems to demonstrate that the focal loss helps to better localize objects of different classes but does not give an equally effective contribution to the classification. It is worth noting that the classification results are computed on the objects actually detected by each method (see Equation (11)), so the approaches that achieved a higher Recall correctly classified more object instances.

We can generally observe that the classification accuracy is around 100% on Cars and Pedestrians, and almost 95% on the Bicycles, while it dramatically drops on the Motorcycle, Bus, Truck and Rider classes. If we consider that these classes are also the least detected (see Table 1), we can conclude that some object categories are not correctly recognized and can cause errors (collisions, steering, decelerations) in the autonomous driving algorithm.

### 4.3. Tracking Results

We firstly analyze the performance of the tracking methods computed with the CLEAR metrics; these results are reported in Table 3. We can note a substantial gap between the first three methods and all the others, especially in terms of mMOTA. These results partially reflect what was observed for the detection, where ConvNext, SwinT and HRNet already demonstrated a remarkable performance; ConvNext is slightly better than the others, probably thanks to the higher detection Recall. The combination of these multi-object detectors with QDTrack, which proves to be the most effective data association method on images acquired on board the vehicle, allows to significantly reduce |FN| and to increase mMOTA (0.41–0.42). The superiority of QDTrack is evident by analyzing the mMOTP value, which measures the average association similarity, of the methods that use it (between 0.83 and 0.89); UniTrack, FairMOT and DeepSORT achieve a substantially lower mMOTP (between 0.74 and 0.78). Not surprisingly, the same result can be observed in terms of IDSW, whose average value (less than 6000) is clearly lower than the other approaches (more than 20,000), demonstrating better stability in object tracking. It is worth noting that, despite what has been observed on QDTrack, not all the methods that use it occupy the first positions. In fact, half of the first 10 positions in the ranking are taken by UniTrack. On one hand, this result points out that the contribution of the multi-object detector on the overall accuracy is significant; on the other hand, it seems that the effectiveness of the data association method also depends on the bounding box provided by the multi-object detector.

The results obtained in terms of IDF1, shown in Table 4, confirm most of the observations deduced from the analysis of the CLEAR metrics. Even with trajectory level associations, ConvNext, SwinT and HRNet with QDTrack achieve the best IDF1 by far (0.54 to 0.56); the contribution of QDTrack is more evident in terms of mIDP (as it was on mMOTP), while multi-object detectors substantially impact mIDR (in the same order as detection Recall). In trajectory-level associations, contrary to what was previously observed, DeepSORT appears to be more effective than UniTrack, especially in terms of mIDR; FairMOT, on the other hand, obtained rather modest results, while on the CLEAR metrics it was better.

ConvNext, SwinT and HRNet with QDTrack demonstrate their effectiveness even in terms of mHOTA, as evident from the results reported in Table 5. ConvNext maintains a slight advantage in the detection capability (mDetA = 0.39), while the association accuracy is the same as SwinT (mAssA = 0.55); the best mHOTA (0.46) is mainly due to the slightly higher detection Recall of the method with respect to SwinT (0.45) and HRNet (0.44). QDTrack occupies the first five positions of the ranking, improving the performance of multi-object detectors not particularly effective in the previous leaderboards, such as YOLOX and RetinaNet; the average of the results on variable values of the localization threshold, between 0.05 and 0.95 with a step of 0.05, seems to favor the stability previously demonstrated by QDTrack. Once again, DeepSORT outperforms UniTrack and FairMOT, with a more marked gap in terms of mAssA, even if the distance from QDTrack remains considerable. We can note the discrete positioning of RetinaNet in all the rankings, which demonstrates the importance of managing the imbalance between the classes, especially adopting metrics that perform associations according to the class.

The results in terms of TETA, reported in Table 6 and based on class-agnostic associations, definitely demonstrate the superiority of QDTrack for the problem at hand. The adopted matching strategy, independent of the class, further favors associations based on similarity and rewards not only of the data association capability of QDTrack, but also the localization effectiveness of the multi-object detectors. ConvNext, SwinT and HRNet occupy the top of the ranking, with the first standing out for its localization capability (LocA = 0.41) and the second for its classification accuracy (ClsA = 0.61). In this case, the superiority over the others is less evident, since YOLOX (TETA = 0.45) and YOLOv5 (TETA = 0.43) are close to the best performance (TETA = 0.49) thanks to their excellent performance in terms of ClsA (0.68 and 0.67), already shown in Section 4.2; the same cannot be observed for LocA, which is a favorable index for almost all methods based on the top three multi-object detectors. In class-agnostic optimization, UniTrack outperforms DeepSORT in almost all the approaches; FairMOT, on the other hand, occupies one of the last positions.

**Figure 4:**
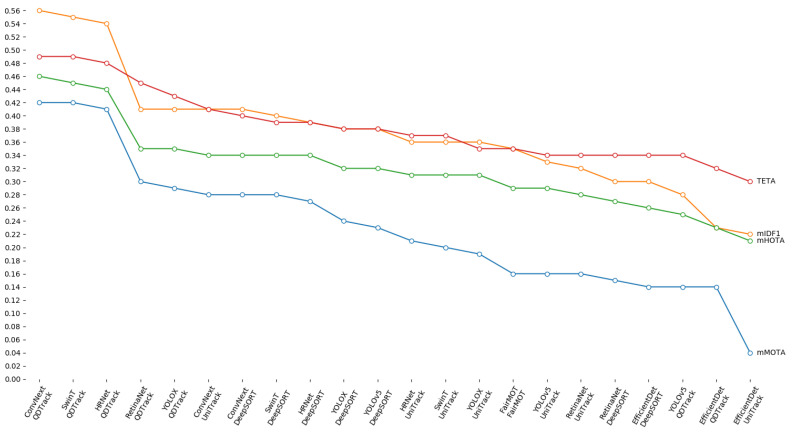
Comparison between the considered methods in terms of mMOTA, mIDF1, mHOTA and TETA. Each line represents a metric whose value is reported on the y-axis, while the methods are scattered on the x-axis. For all the metrics, the superiority of ConvNext, SwinT and HRNet combined with QDTrack is evident.

A summary representation of all the results described above is shown in Figure 4. By observing the chart, the superiority of the methods that use ConvNext, SwinT or HRNet as detector and QDTrack as tracker is even more evident. Indeed, for each metric we can note a substantial gap between these three methods and all the others, clearly visible as a high step. This evidence confirms what has been discussed so far.

## 5. Discussion

**Figure 5:**
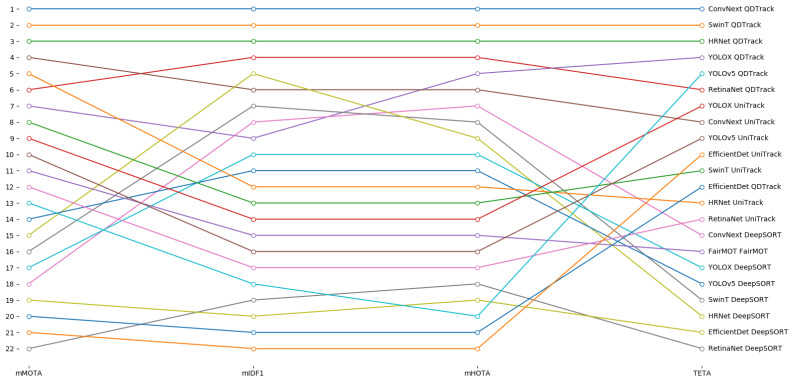
Ranking of the considered methods in terms of mMOTA, mIDF1, mHOTA and TETA. Each line represents a method whose position in the ranking is reported on the y-axis, while the metrics are scattered on the x-axis. ConvNext, SwinT and HRNet combined with QDTrack always keep the top three positions.

The analysis of the experimental results allows the discussion of interesting insights. On one hand, the adoption of QDTrack with a multi-object detector among ConvNext, SwinT or HRNet is the most effective approach for multi-object tracking in autonomous driving scenarios, as evident from the ranking reported in Figure 5. On the other hand, the absolute performance should be substantially improved, even for the best methods. Indeed, from Table 1 we can observe that 15% of the cars ($Re_{C}=0.85$), 20% of the buses ($Re_{Bus}≃0.80$) and 23% of the trucks ($Re_{T}≃0.77$) are not detected by ConvNext and SwinT; in addition, around 25% of the pedestrians ($Re_{P}≃0.75$) and 40% of bicycles and motorcycles ($Re_{M}≃0.65$ and $Re_{B}≃0.58$) are invisible for the considered detectors. Also for the riders, we can note a miss rate around 25% ($Re_{R}≃0.75$); in addition, the analysis of the confusion matrix of ConvNext allows us to observe that about 35% of the riders are misclassified as pedestrians. This confusion can cause serious errors in terms of collisions, steering and decelerations. Furthermore, the results in Table 2 point out the wrong classification of a high percentage of the objects belonging to less-represented classes, even when they are correctly detected. Therefore, there is certainly room for improvement for multi-object detectors applied in autonomous driving scenarios.

The issues described above may be less serious if the missed detections and wrong classifications take place for objects that are far from the vehicle. Indeed, depending on the speed of the vehicle, objects far away may not require any braking or steering action. Nevertheless, a drawback of current tracking metrics is that they do not consider distance from objects and thus do not weight the errors according to that distance. Therefore, we have no way to evaluate this aspect, which could instead be relevant for preferring one method to another. A future research direction should be the definition of metrics that take into account the distance from objects and, consequently, the adaptation of loss functions to train multi-object detectors and the neural networks used to extract object descriptors for appearance-based re-identification. Obviously, in support of this research direction, it would be useful to have data annotated also with the distance from the objects, which are available, for example, in Waymo Open Dataset; alternatively, the only possibility would be to estimate the distance from the object, starting from the extrinsic and the intrinsic matrices of the camera and the class of the object.

It is also worth noting that none of the multi-object tracking methods have been optimized to perform data association on multi-class objects; in fact, all the considered approaches are designed to carry out the re-identification of a single class of objects, typically people, and are only re-adapted for the multi-class problem at hand. In addition, in the case under examination, the camera is moving and the objects (especially the vehicles parked on the roadside) are framed from different angles and can have totally different frame-by-frame appearances. Therefore, another research direction could be to design data association algorithms that take into account the different classes of objects of interest and the peculiarities of the images acquired by the vehicle on the road (especially occlusions and different points of view). Similarly, new methods for predicting the next position occupied by tracked objects should be investigated; such approaches could make use of an ego-motion estimation based on image analysis, or even better, on information collected by the inertial navigation system of the vehicle.

Finally, another very serious shortcoming is the lack of simulations of such errors (detection and/or tracking) in driving scenarios in order to evaluate their impact on autonomous navigation. To this purpose, it would be useful to have a framework in which artificial vision algorithms can be integrated and the negative effects of perception errors on autonomous navigation can be verified in simulation. This integration can be done within a simulator such as CARLA, in which it is possible to implement a navigation algorithm that exploits the considered multi-object tracking methods in its perception module to estimate position, speed and orientation of cars, trucks, buses, pedestrians, bicycles and motorbikes. Furthermore, it is necessary to define an experimental framework that allows to evaluate the performance of the various multi-object tracking methods. On one hand, it is possible to compute the standard multi-object tracking metrics, or any additional new metric, by comparing the estimated perceptions with the groundtruth provided by the simulator. On the other hand, it is required to compute a driving score that takes into account, for example, collisions due to false negatives or incorrect estimation of the position of the objects which may be evaluated, as well as unnecessary decelerations or sharp steering due to false positives. The evaluation may also consider the number of infractions, the lane invasions, the number of collisions, the driving comfort, the navigation time and so on. This framework would produce results that allow to evaluate both the tracking performance and the effect of errors on the autonomous navigation. It is not easy to develop such a framework, as the navigation algorithm performs its operations in the world reference system; therefore, its realization requires the definition of multi-sensor fusion algorithms for obtaining 3D coordinates, ego-state and ego-motion estimation methods, and transformation of the tracking results into estimation of the speed and orientation of vehicles and pedestrians on the road. Providing such a tool would be a great contribution to the development of research in this field.

## 6. Conclusions

In this paper, we have proposed a framework for evaluating multi-object detection and tracking methods on image sequences acquired by a camera installed on board an autonomous driving vehicle. Twenty-two different approaches were trained and tested on the BDD100K dataset, computing several evaluation metrics measuring detection, classification and tracking capability of the methods. The experimental analysis allowed to highlight the superiority of QDTrack as a tracking method, observing that it performs best in combination with a detector based on ConvNext, SwinT or HRNet; such multi-object tracking methods had never before been applied in autonomous driving. The proposed framework has allowed for a deeper evaluation of the contribution provided by each of the modules on the overall performance of the methods. Furthermore, the discussion of the experimental results brought out the shortcomings of the currently existing approaches and allowed to outline possible future directions of research in this field. In particular, we conclude that: (i) object detectors and their classification branches definitely need to be improved to ensure higher reliability on all the classes of interest; (ii) the metrics used to evaluate the multi-object tracking methods in road scenarios and the loss functions used in the learning procedure must consider specific aspects of this field, such as the class of the objects and their distance from the vehicle; (iii) the data association methods must be designed to solve a multi-class object re-identification problem with a moving camera, thus investigating appearance-based and position-based similarity functions that take these aspects into account; (iv) the navigation algorithms that make use of the aforementioned multi-object tracking methods must be simulated in order to verify the impact of perception errors in terms of collisions and sudden steerings and decelerations.

## Figures and Tables

**Table 1:** Detection results computed with α=0.5 in terms of Precision (Pr), Recall (Re), F1-score (F1), mIoU and Recall for each class. The methods are ordered for descending F1-score.

	Pr	Re	F1	mIoU	ReP	ReR	ReC	ReT	ReBus	ReM	ReB
SwinT	0.73	0.82	0.78	0.84	0.74	0.70	0.85	0.76	0.80	0.63	0.58
ConvNext	0.71	0.83	0.77	0.84	0.75	0.71	0.85	0.78	0.81	0.67	0.58
HRNet	0.74	0.81	0.77	0.83	0.72	0.71	0.84	0.76	0.79	0.66	0.57
YOLOX	0.75	0.79	0.77	0.84	0.68	0.64	0.83	0.71	0.73	0.59	0.53
YOLOv5	0.74	0.78	0.76	0.84	0.65	0.56	0.81	0.71	0.72	0.60	0.55
FairMOT	0.93	0.56	0.70	0.80	0.43	0.30	0.60	0.48	0.42	0.15	0.20
EfficientDet	0.71	0.64	0.67	0.82	0.45	0.41	0.69	0.57	0.57	0.47	0.30
RetinaNet	0.58	0.74	0.65	0.81	0.62	0.60	0.77	0.67	0.71	0.51	0.48

**Table 2:** Classification results in terms of overall accuracy (*A*) and accuracy for each class (Pedestrian, Rider, Car, Truck, Bus, Motorcycle, Bicycle). The methods are ordered for descending *A*.

	*A*	$A_{P}$	$A_{R}$	$A_{C}$	$A_{T}$	$A_{\mathrm{Bus}}$	$A_{M}$	$A_{B}$
ConvNext	0.97	0.99	0.68	1.00	0.68	0.78	0.78	0.95
SwinT	0.97	0.99	0.67	1.00	0.68	0.79	0.79	0.94
YOLOX	0.97	0.99	0.65	1.00	0.66	0.79	0.81	0.94
YOLOv5	0.97	0.99	0.65	1.00	0.66	0.78	0.74	0.95
HRNet	0.97	0.99	0.65	0.99	0.68	0.76	0.79	0.94
FairMOT	0.97	1.00	0.54	1.00	0.66	0.76	0.94	0.95
EfficientDet	0.97	0.99	0.55	1.00	0.59	0.74	0.79	0.91
RetinaNet	0.96	0.97	0.52	0.99	0.58	0.68	0.72	0.88

**Table 3:** Tracking results on the BDD100K validation set in terms of mMOTA and mMOTP. We also report |FN|, |FP| and IDSW to analyze the data association capability of the methods. The approaches are ranked for descending mMOTA.

Detector	Tracker	mMOTA	mMOTP	|FN|	|FP|	IDSW
ConvNext	QDTrack	0.42	0.83	137,827	19,160	7262
SwinT	QDTrack	0.42	0.83	141,444	17,215	5222
HRNet	QDTrack	0.41	0.83	135,707	20,572	5585
ConvNext	UniTrack	0.30	0.77	180,729	22,821	38,772
HRNet	UniTrack	0.29	0.77	188,400	22,706	35,820
RetinaNet	QDTrack	0.28	0.83	207,003	19,715	8326
YOLOX	QDTrack	0.28	0.86	229,427	5043	4051
SwinT	UniTrack	0.28	0.77	207,309	16,980	21,608
YOLOX	UniTrack	0.27	0.78	210,391	11,065	32,710
YOLOv5	UniTrack	0.24	0.79	235,107	7168	27,355
FairMOT	FairMOT	0.23	0.77	190,537	21,262	43,140
RetinaNet	UniTrack	0.21	0.77	234,119	19,524	30,992
YOLOv5	QDTrack	0.20	0.89	254,542	3823	2665
YOLOv5	DeepSORT	0.19	0.75	183,116	63,681	18,814
HRNet	DeepSORT	0.16	0.74	156,518	98,360	23,299
SwinT	DeepSORT	0.16	0.74	156,647	98,523	23,307
YOLOX	DeepSORT	0.16	0.75	165,816	76,382	21,065
ConvNext	DeepSORT	0.15	0.74	153,056	103,642	22,995
EfficientDet	DeepSORT	0.14	0.77	272,843	36,474	9998
EfficientDet	QDTrack	0.14	0.87	305,060	5244	2379
EfficientDet	UniTrack	0.14	0.80	314,947	3784	15,085
RetinaNet	DeepSORT	0.04	0.74	199,032	95,992	22,230

**Table 4:** Tracking results on the BDD100K validation set in terms of mIDF1. We also report mIDR and mIDP to analyze the data association sensitivity and specificity of the methods. The approaches are ranked for descending mIDF1.

Detector	Tracker	mIDF1	mIDR	mIDP
ConvNext	QDTrack	0.56	0.43	0.82
SwinT	QDTrack	0.55	0.42	0.83
HRNet	QDTrack	0.54	0.41	0.82
HRNet	DeepSORT	0.41	0.34	0.52
SwinT	DeepSORT	0.41	0.34	0.52
RetinaNet	QDTrack	0.41	0.29	0.76
ConvNext	UniTrack	0.41	0.30	0.67
ConvNext	DeepSORT	0.40	0.33	0.51
YOLOX	QDTrack	0.39	0.27	0.83
YOLOv5	DeepSORT	0.38	0.29	0.58
YOLOX	DeepSORT	0.38	0.30	0.54
HRNet	UniTrack	0.36	0.27	0.61
SwinT	UniTrack	0.36	0.26	0.66
YOLOX	UniTrack	0.36	0.26	0.69
FairMOT	FairMOT	0.35	0.24	0.69
YOLOv5	UniTrack	0.33	0.22	0.74
RetinaNet	UniTrack	0.32	0.22	0.63
RetinaNet	DeepSORT	0.30	0.24	0.43
YOLOv5	QDTrack	0.30	0.20	0.88
EfficientDet	DeepSORT	0.28	0.19	0.62
EfficientDet	QDTrack	0.23	0.15	0.82
EfficientDet	UniTrack	0.22	0.14	0.74

**Table 5:** Tracking results on the BDD100K validation set in terms of mHOTA. We also report mDetA and mAssA to analyze the detection and association capabilities of the methods. The reported are obtained by varying the localization threshold from 0.05 to 0.95 with a step of 0.05. The approaches are ranked for descending mHOTA.

Detector	Tracker	mHOTA	mDetA	mAssA
ConvNext	QDTrack	0.46	0.39	0.55
SwinT	QDTrack	0.45	0.38	0.55
HRNet	QDTrack	0.44	0.37	0.54
YOLOX	QDTrack	0.35	0.27	0.47
RetinaNet	QDTrack	0.35	0.28	0.44
ConvNext	DeepSORT	0.34	0.27	0.44
SwinT	DeepSORT	0.34	0.28	0.45
HRNet	DeepSORT	0.34	0.27	0.45
ConvNext	UniTrack	0.34	0.30	0.40
YOLOv5	DeepSORT	0.32	0.25	0.44
YOLOX	DeepSORT	0.32	0.25	0.44
YOLOX	UniTrack	0.31	0.27	0.39
HRNet	UniTrack	0.31	0.29	0.34
SwinT	UniTrack	0.31	0.26	0.37
FairMOT	FairMOT	0.29	0.24	0.39
YOLOv5	UniTrack	0.29	0.23	0.39
RetinaNet	UniTrack	0.28	0.23	0.35
RetinaNet	DeepSORT	0.27	0.21	0.38
EfficientDet	DeepSORT	0.26	0.17	0.40
YOLOv5	QDTrack	0.25	0.17	0.38
EfficientDet	QDTrack	0.23	0.14	0.39
EfficientDet	UniTrack	0.21	0.14	0.34

**Table 6:** Tracking results on the BDD100K validation set in terms of TETA. We also report LocA, AssocA and ClsA to analyze localization, association and classification capabilities of the methods. The approaches are ranked for descending TETA.

Detector	Tracker	TETA	LocA	AssocA	ClsA
ConvNext	QDTrack	0.49	0.41	0.46	0.60
SwinT	QDTrack	0.49	0.39	0.46	0.61
HRNet	QDTrack	0.48	0.39	0.46	0.59
YOLOX	QDTrack	0.45	0.26	0.42	0.67
YOLOv5	QDTrack	0.43	0.21	0.40	0.68
RetinaNet	QDTrack	0.41	0.29	0.36	0.57
YOLOX	UniTrack	0.40	0.27	0.33	0.59
ConvNext	UniTrack	0.39	0.32	0.32	0.53
YOLOv5	UniTrack	0.39	0.22	0.33	0.60
EfficientDet	UniTrack	0.38	0.14	0.34	0.65
SwinT	UniTrack	0.38	0.28	0.32	0.54
EfficientDet	QDTrack	0.37	0.15	0.35	0.61
HRNet	UniTrack	0.37	0.31	0.30	0.52
ConvNext	DeepSORT	0.35	0.39	0.37	0.29
RetinaNet	UniTrack	0.35	0.24	0.29	0.51
SwinT	DeepSORT	0.34	0.37	0.36	0.29
HRNet	DeepSORT	0.34	0.37	0.35	0.29
YOLOv5	DeepSORT	0.34	0.31	0.36	0.35
YOLOX	DeepSORT	0.34	0.35	0.36	0.32
FairMOT	FairMOT	0.34	0.30	0.28	0.46
EfficientDet	DeepSORT	0.32	0.20	0.35	0.41
RetinaNet	DeepSORT	0.30	0.31	0.34	0.26

## Data Availability

Data sharing not applicable.
